# Osteoid osteoma: diagnosis and treatment

**DOI:** 10.1590/0100-3984.2015.48.4e1

**Published:** 2015

**Authors:** Clarissa Canella

**Affiliations:** 1 MD, Radiologist, Clínica de Diagnóstico por Imagem (CDPI), Rio de Janeiro, RJ, Brazil. E-mail: clacanella@yahoo.com.br.

Osteoid osteoma is the third most common benign neoplasm of bone, occurring predominantly in young, male patients^(1)^. In most of cases, the patients report inflammatory-like pain that worsens at night and is alleviated with the use of nonsteroidal anti-inflammatory drugs. Osteoid osteoma is classically characterized at conventional radiography or computed tomography as a well defined lytic area representing the vascularized central nidus, surrounded by sclerosis and cortical thickening^(2-4)^. Computed tomography is an excellent imaging method to identify the central nidus of the tumor and can also be utilized for treatment, as a guidance for percutaneous removal of the nidus, as described by the article developed by Petrilli et al.^(5)^ and published in the present issue of **Radiologia Brasileira**. The authors have evaluated computed tomography-guided percutaneous trephine removal of the nidus in 18 cases of osteoid osteoma, demonstrating that this is a safe and effective method for surgical resection of the lesion with reduced hospitalization time and less postoperative pain.

It is important observe that in some cases, the osteoid osteoma nidus cannot be appropriately individualized at conventional imaging methods such as radiography and computed tomography, which makes the diagnosis more difficult^(6-8)^. In such cases, magnetic resonance imaging, that has already been discussed in recent studies published by the Brazilian literature^(9-12)^, may be utilized as an aid in the differential diagnosis, particularly by means of inphase, out-of-phase and perfusion-weighted imaging of the lesion.

Considering that most tumors tend to replace the fatty and hematopoietic marrow components, the nidus of the osteoid osteoma may present with persistent signal intensity at out-of-phase sequences as compared with in-phase images^(13)^. On the other hand, the decrease in signal intensity at out-of-phase sequences as compared with in-phase images would indicate the presence of fat and water in the bone marrow, which would indicate a lower probability of the presence of a neoplasm^(13)^.

Perfusion-weighted imaging has also been quite useful in the identification of the nidus of osteoid osteomas and in the diagnosis of the tumor. Most osteoid osteomas present with early and intense gadolinium enhancement, with a washout-type curve (type IV) resulting from the nidus hypervascularization^(9,10)^.

Finally, some imaging methods can be utilized in the investigation of osteoid osteomas. Computed tomography plays a fundamental role in the identification of the nidus of the lesion as well as in the percutaneous removal of the nidus, as didactically demonstrated by Petrilli et al.^(5)^. In some cases where the nidus cannot be appropriately identified, making the differential diagnosis more difficult, magnetic resonance imaging may be utilized as a useful tool, particularly by means of in-phase, out-of-phase and perfusion-weighted imaging of the lesion.

## References

[1] Greenspan A (1993). Benign bone-forming lesions: osteoma, osteoid osteoma, and
osteoblastoma. Clinical, imaging, pathologic, and differential
considerations. Skeletal Radiol.

[2] Woods ER, Martel W, Mandell SH (1993). Reactive soft-tissue mass associated with osteoid osteoma: correlation
of MR imaging features with pathologic findings. Radiology.

[3] Goldman AB, Schneider R, Pavlov H (1993). Osteoid osteomas of the femoral neck: report of four cases evaluated
with isotopic bone scanning, CT, and MR imaging. Radiology.

[4] Assoun J, Richardi G, Railhac JJ (1994). Osteoid osteoma: MR imaging versus CT. Radiology.

[5] Petrilli M, Senerchia AA, Petrilli AS (2015). Computed tomography-guided percutaneous trephine removal of the nidus
in osteoide osteoma patients: experience of a single center in
Brazil. Radiol Bras.

[6] Biebuyck JC, Katz LD, McCauley T (1993). Soft tissue edema in osteoid osteoma. Skeletal Radiol.

[7] Ehara S, Rosenthal DI, Aoki J (1999). Peritumoral edema in osteoid osteoma on magnetic resonance
imaging. Skeletal Radiol.

[8] Costa FM, Canella C, Gasparetto E (2011). Advanced magnetic resonance imaging techniques in the evaluation of
musculoskeletal tumors. Radiol Clin North Am.

[9] Nakamura SA, Lorenzato MM, Engel EE (2013). Incidental enchondromas at knee magnetic resonance imaging:
intraobserver and interobserver agreement and prevalence of imaging
findings. Radiol Bras.

[10] Souza CG, Gasparetto EL, Marchiori E (2013). Pyogenic and tuberculous discitis: magnetic resonance imaging findings
for differential diagnosis. Radiol Bras.

[11] Machado BB, Lima CMAO, Junqueira FP (2013). Magnetic resonance imaging in intersection syndrome of the forearm:
iconographic essay. Radiol Bras.

[12] Terazaki CRT, Trippia CR, Trippia CH (2014). Synovial chondromatosis of the shoulder: imaging
findings. Radiol Bras.

[13] Zajick DC, Morrison WB, Schweitzer ME (2005). Benign and malignant processes: normal values and differentiation with
chemical shift MR imaging in vertebral marrow. Radiology.

